# Amyotrophic lateral sclerosis mimic syndromes

**Published:** 2016-04-03

**Authors:** Majid Ghasemi

**Affiliations:** ^1^ Neuroscience Research Center AND Department of Neurology, School of Medicine, Isfahan ‎University of Medical Sciences, Isfahan, Iran

**Keywords:** Amyotrophic Lateral Sclerosis, Motor Neuron Disease, Differential Diagnosis

## Abstract

Amyotrophic lateral sclerosis (ALS) misdiagnosis has many broad implications for the patient and the neurologist. Potentially curative treatments exist for certain ALS mimic syndromes, but delay in starting these therapies may have an unfavorable effect on outcome. Hence, it is important to exclude similar conditions. In this review, we discuss some of the important mimics of ALS.

## Introduction

Amyotrophic lateral sclerosis (ALS) is a progressive and almost always devastating neurodegenerative disorder. It is a kind of a heterogeneous group of disorders known as motor neuron diseases (MNDs). The most common MND of adults is ALS. The prototypic form of this lethal disorder simultaneously involves both upper motor neuron (UMN) and lower motor neuron (LMN) which progresses from a region of neuraxis to others and final death, typically from respiratory involvement.^1^

In populations of European origin, ALS is the more common in men than in women (1.2-1.5:1).^2^ On the other hand, most studies show that bulbar-onset ALS displays a female predominance.^3^^,^^4^ In contrast to other neurodegenerative disorders, the risk of developing ALS peaks between the ages of 50 and 75 years, and declines thereafter.^2^^,^^5^ This feature suggests that aging is not a single risk factor of ALS. The incidence of sporadic ALS is reported to be between 2.16 per 100000 person years population (average 1.89 per 100000/year), with a uniform incidence across Europe. It is estimated that the general risk of ALS for lifetime is 1:400 for women and 1:350 for men. Incidence decreases quickly after age of 80 years.^2^ The Research Group of World Federation of Neurology on MNDs have build up the “El Escorial” diagnostic criteria in 1994.^6^ Moreover, the revised in 2000 (Airlie House Criteria)^7^ to aid in diagnosing and classifying ALS patients specially for research studies (Tables 1 and 2).

In the early stages of disease patients are most likely to benefit from treatment, but these criteria may have low sensitivity to do definite diagnosis. Because of these limitations, the criteria have been modified to help early diagnosis and to optimize levels of diagnostic certainty.^8^^-^^11^

It is important to rule out treatable mimics. ALS misdiagnosis has many broad implications for the patient and the neurologist. Potentially curative treatments exist for certain ALS mimic syndromes, but delay in starting these therapies may have an unfavorable effect on the outcome.

The term ALS mimic syndrome has been used to describe a heterogeneous group of conditions that their presentation and clinical features may resemble those of ALS at the beginning.

**Table 1:** Diagnostic criteria for amyotrophic lateral sclerosis (ALS)

The diagnosis of ALS requires the presence of (positive criteria)
LMN signs (including EMG features in clinically unaffected muscles)
UMN signs
Progression of symptoms and signs
The diagnosis of ALS requires the absence of (diagnosis by exclusion)
Sensory signs
Sphincter disturbances
Visual disturbances
Autonomic features
Basal ganglion dysfunction
Alzheimer-type dementia
ALS “mimic” syndromes
The diagnosis of ALS is supported by
Fasciculations in one or more regions
Neurogenic changes in EMG results
Normal motor and sensory nerve conduction
Absence of conduction block

ALS: Amyotrophic lateral sclerosis; EMG: Electromyography; UMN: Upper motor neuron; LMN: Lower motor neuron

It is different to ALS with laboratory abnormalities of uncertain significance, which is a subgroup of ALS that occurs in association with a defined laboratory abnormality that is of doubtful implication to the pathogenesis of ALS.^11^

To our knowledge, there have been few published studies of ALS mimic syndromes.^12^^,^^13^ Population-based studies have shown that almost 10% of patients with ALS diagnosis have had another disease.^14^

**Mimics**

Approach to the differential diagnosis of ALS (ALS mimic syndromes) can be in terms of the anatomy, symptoms, or clinical presentation. Here, we discuss mimics based on the nervous system anatomy.

**Brain**

Adult polyglucosan body disease (APBD) is a late-onset, slowly progressive disorder of both UMN and LMN, like ALS, but it has other neurologic sings such as cognitive decline, distal sensory loss, and disturbances of bladder and bowel function. Magnetic resonance imaging of the brain may reveal diffuse white-matter signal increase on T_2_-weighted images. The diagnosis is confirmed by the finding of characteristic pathological changes in samples from peripheral nerve, cerebral cortex, spinal cord, or skin. There are non-membrane-bound periodic acid-Schiff positive cytoplasmic polyglucosan bodies in axons and neural sheath cells.

Mutations of the glycogen-branching enzyme (GBE) gene are the cause of this disorder in Ashkenazi Jewish patients, but APBD occurs in many different populations, and considerable molecular heterogeneity has been noted, with otherwise typical cases lacking GBE mutations despite deficiency of enzyme activity.^15^^,^^16^

**Brainstem and spinal cord**

Adrenomyeloneuropathy present with spastic paraparesis, areflexia, sphincter disturbance, and sensory loss. It is a peroxisomal disorder caused by a defect in beta-oxidation of very long-chain fatty acids, presenting in their third or fourth decade of life. Increased plasma levels of very long-chain fatty acids make the diagnosis.^17^

**Table 2:** El Escorial World Federation of Neurology criteria for diagnosis of amyotrophic lateral sclerosis (ALS)

Clinically definite ALS
UMN and LMN clinical signs or electrophysiological evidence in three regions
Clinically definite ALS-laboratory supported
UMN and/or LMN clinical signs in one region and the patient is a carrier of a pathogenic SOD1-gene mutation
Clinically probable ALS
UMN and LMN clinical or electrophysiological evidence by LMN and UMN signs in two regions with some UMN signs rostral to the LMN signs
Clinically possible ALS
UMN and LMN clinical or electrophysiological signs in one region only, or
UMN signs in at least two regions, or
UMN and LMN signs in two regions with no UMN signs rostral to LMN signs. Neuroimaging and laboratory studies have excluded other diagnoses.

ALS: Amyotrophic lateral sclerosis; LMN: Lower motor neuron; UMN: Upper motor neuron, SOD1: Superoxide dismutase 1

In multiple sclerosis a both of UMN and LMN involvement may be seen in the setting of plaque formation at root exit zones, combined with central nervous system (CNS) lesions. Lesions at the level of foramen magnum and medulla such as infarct, syrinx, demyelination, and neoplasm, may suggest the bulbar-onset ALS, so neuroimaging may be essential in the evaluation of suspected cases of ALS.

Syringomyelia may present with atrophy and weakness, but a characteristic pattern of dissociated sensory loss typically occurs, and the disease progresses at a much slower rate in a usually younger patient than ALS. Another consideration is vitamin B12 deficiency, but prominent sensory findings usually distinguishes it from ALS.

However, patients occasionally may lack sensory findings, so it is prudent to routinely measure a vitamin B12 level to exclude this treatable condition.

Allgroveor “Four-A” syndrome, a rare autosomal recessive disorder that derives its name from the combination of achalasia, alacrima, adrenal insufficiency, and amyotrophy.

It can manifest from the first decade of life with dysphagia and adrenal insufficiency and a broad range of neurological problems in later life.

A particular phenotype like ALS including pyramidal features and LMN involvement has been described in this disease.^18^

The upper-limb amyotrophy, with predominance on the ulnar side of the hands, resembles that of ALS, and bulbar sign and symptoms (tongue atrophy and fasciculation, etc.) have led to the misdiagnosis of bulbar ALS.^19^

The proximity of both UMN and LMN structures in the cervical spine, makes degenerative myeloradiculopathy an important diagnostic challenge in cases of suspected ALS. 

On the other hand, incidental finding of cervical spondylosis is highly prevalent in ALS patients.^20^ Symptoms such as emotional lability and abnormal signs of the cranial region are supportive in differentiate neck pathology from ALS. Furthermore, in contrast to ALS, cervical spondylosis is unlikely to cause LMN signs in the hand muscles or widespread fasciculations.^21^

Specially, the presence of fasciculations in the bulbar and lumbosacral areas would be in contrast to the diagnosis of neck pathology.

Pure motor manifestations and without sphincteric dysfunction are not rare in patients with cervical spondylotic myelopathy, which may be similar to the clinical manifestations of ALS, using such terms as dissociated motor loss or cervical spondylotic amyotrophy.

The pathogenesis of this syndrome may be selective damage to the ventral root due to compresssion by posterolateral osteophytes; on the other hand, vascular insufficiency of the anterior horn cells may be caused by dynamic cord compression. This condition is characterized by segmental muscular atrophy and neurogenic electromyography (EMG) changes, which may be multi-segmental but not as diffuse as is found in ALS.^21^ Thus, we have to consider compressive radiculopathy as a cause of focal LMN signs in a limb. Furthermore, other causes of polyradiculopathies such as neuplasms (lymphoma or leukemia), radiation, and infections (viral and spirochetal) may mimic ALS.

In the differential diagnosis of slowly evolving spastic paraparesis, we have to consider hereditary spastic paraparesis. However, this disorder is differentiated by a family history together with very slow progression, sphincteric disturbance, and an absence of LMN, bulbar, and respiratory involvement.^21^

**Anterior Horn Cell**

Kennedy’s disease, is an X-linked disorder of brainstem and spinal cord LMNs and classically presents in the third or fourth decade in males with atrophy and weakness of bulbar, facial, and limb girdle muscles; tremor; perioral fasciculations; mild cognitive impairment; sensory disturbance; and signs of endocrine dysfunction such as diabetes mellitus, gynecomastia, and testicular atrophy.^22^^,^^23^

In addition of above-mentioned features, a moderately increased creatine kinase (CK) and low amplitude sensory nerve action potential (SNAPs) can help to differentiate it from ALS. To confirm the diagnosis, a genetic test for detection of CAG repeat expansion of the androgen receptor gene is recommended.

Hexosaminidase A (Hex-A) is a lysosomal enzyme that contributes in the degradation of the ganglioside GM2. Accumulation of GM2 leads to degeneration of nerve cells and produces a wide spectrum of neurological disorders. Total deficiency produces a fatal infantile disorder, Tay-Sachs disease. Partial deficiency of enzyme activity causes a variety of adult-onset neurological disorders, characterized by combined involvement of UMN and LMNs, cerebellar and extrapyramidal dysfunction, and psychosis or dementia.^23^ It is commonly cited in the differential diagnosis of ALS, especially in atypical cases. In this disorder, EDX studies may reveal prominent complex repetitive discharges on needle EMG and abnormal SNAPs.

Benign monomelic amyotrophy is another differential diagnosis, specially mimicing monomelic-onset ALS. It typically presents as focal atrophy and weakness of one limb, or part thereof, without sensory dysfunction, predominantly in second and third decades of young men. Fasciculations are prominent and reflexes may be either reduced or normal. It may progress for a few years with eventual stabilization. Needle EMG may reveal relatively sparse fibrillation potentials (in contrast to ALS) in affected muscles along with neurogenic motor unit action potentials (MUAPs) in both clinically affected and unaffected limbs.

Lymphoma may present subacutely with LMN manifestations typically in the lower extremities. Rarely lymphoma may present with a combination of both UMN and LMN signs, similar to ALS. Apart from lymphoma, Waldenstrom’s macroglobulinemia and myeloma may be present by MND.

Paraneoplastic encephalomyelitis may present with motor neuron disorder alone, like ALS, and sensory and autonomic features and ataxia occur later. Associated anti-neuronal antibodies, may be detected. The anti-amphiphysin presentation is usually PLS like, but unlike true PLS it rapidly deteriorates. The anti-Ma associated disorder varies but can be like progressive muscular atrophy.^24^ The association of ALS with solid malignancy is rather unclear.

Radiation toward the retroperitoneal area or spinal region can cause a pure LMN syndrome in the lumbosacral segment, simulating LMN onset ALS. It may appear many years after the irradiation. Myokymic discharges and non-resolving conduction blocks are distinguishing electrodiagnostic (EDX) features.^25^

Focal muscle weakness and wasting in post-polio syndrome progresses slowly to other regions over many years, and in contrast to ALS, it does not usually cause death. Moreover, it does not involve UMNs.^26^

**Peripheral neuropathies**

Multifocal motor neuropathy with conduction block is another mimic of ALS. It presents by onset of focal motor weakness, usually in a distal upper extremity, accompanied commonly by fasciculations and cramps. It has a male predominance (3:1) younger age onset (mean 40 years), with no cases reported over age 70 years. 

It is slowly progressive, usually over months or even years. An important clue to the diagnosis is the absence of muscle atrophy despite very significant weakness, until late in the disease course. In addition to above diagnostic clues, anti GM1 antibody and conduction block on nerve conduction study can differentiate this disorder.

**Neuromuscular transmission (NMT) disorders**

The most common NMT disorder in the context of isolated or near isolated bulbar dysfunction is myasthenia gravis (MG). MG is occasionally misdiagnosed as MND and viz. Muscle fatigue, although considered a characteristic feature of MG, occurs in patients with other neuromuscular disorders including MND. EDX study might not be diagnostic of MND in bulbar onset disease but should help to exclude primary NMT disorders, such as MG. Cholinesterase inhibitors used for the treatment of MG can provide transient symptom relief in MND.^26^

**Muscle disorders**

Oculopharyngeal muscular dystrophy may simulate bulbar-onset ALS, but in contrast to ALS, it usually involves the muscles of eyelids and extraocular. In those rare cases that present with bulbar manifestations and subtle or no extraocular involvement, a muscle biopsy may be required to differentiate it from MND. 

Another attractive disorder is isolated neck extensor myopathy, which presents in older persons with dropped head and is associated with signs of active denervation in cervical paraspinal muscles like MND, but the weakness does not spread to other regions.

Because of distal muscle involvement, painless asymmetric weakness, and difficulty swallowing, inclusion body myositis (IBM) may mimic ALS. However, fasciculations and UMN signs obviously are absent. A raised serum CK beyond reasonable titers for denervation (> 1000 IU/L) may be a laboratory clue although it may be normal. In addition to phenotypical similarities, EMG may show neurogenic MUAPs with fibrillation potentials as seen in ALS.

Hence, it may be needed to perform muscle biopsy to confirm IBM by the presence of rimmed vacuoles and intranuclear inclusions.

**Systemic disease**

Hyperthyroidism may misdiagnoses as ALS. It presents with corticospinal tract signs (hyperreflexia), fasciculations, weight loss, and weakness. However, there usually are additional systemic signs such as heat intolerance, anxiety, tremor, tachycardia, and insomnia. It is prudent to include a thyroid function assay in the screening evaluation of ALS patients (Table 3). Weakness may be seen in hyperparathyroidism and mimic LMN onset ALS. Human immunodeficiency virus (HIV) infection may also clinically mimic ALS. A retrospective review of 1700 cases of HIV positive patients with neurological symptoms documented six cases presenting as an ALS-like syndrome.^27^

In each case, antiretroviral therapy was beneficial either in stabilizing or curing the disease. Overall, patients were younger than the typical ALS patients, by signs and symptoms of UMN and LMN involvement, and onset was characteristically in a monomelic pattern followed by rapid spread to other regions over a period of weeks.

Benign fasciculations usually occur under the age of 30 years, with a relapsing-remitting course over a period of months or years. It does not have any other neurologic abnormalities. They occur in a wide variety of disorders and are frequent in the normal population.

Fasciculations with MND typically is asymptomatic and do not recognize until detected by the physician. They are diffuse and rarely are the presenting symptom. It is in contrast to benign fasciculations.

Muscle fasciculation without weakness should be considered a benign phenomenon, although follow-up (sometimes 6 months or more), might be required to confirm benign nature of that.

Needle EMG has distinguishing features that can differentiate benign from MND-associated fasciculations.

The latter tend to have a complex waveform (neurogenic MUAP), may be induced by joint displacement, and are associated with other EDX features of a widespread disorder of anterior horn cells.^28^^-^^36^

**Table 3:** Summary of amyotrophic lateral sclerosis (ALS)‎ differential diagnosis

**Anatomical location of disorder**	**Disease**	**Clinical clues**
CNS ± PNS	Spinocerebellar ataxia type 3	Prominent extrapyramidal and oculomotor signs
	Multiple system atrophy	Ataxia, dysautonomia, sphincter disturbance, and oculomotor disturbances
	Parkinson’s disease	Tremor and response to levodopa
	APBD	Cognitive decline, distal sensory loss, and disturbances of bladder and bowel function
	Hex-A deficiency	Cerebellar ataxia, cognitive deterioration, EDX studies may reveal prominent complex repetitive discharges and abnormal SNAPs
	Allgrove syndrome	Achalasia, alacrima, adrenocorticotrophic insufficiency, and a broad range of neurological problems
Brainstem and spinal cord	Kennedy’s disease	Mild cognitive impairment; sensory disturbance; and signs of endocrine dysfunction
	Cervical spondylosis	Prominent neck pain especially with sphincter involvement
	Adrenomyeloneuropathy	Increased serum VLCFA, sphincter disturbance, sensory loss
	Hereditary spastic paraparesis	family history, very slow progression, sphincter disturbance, absence of LMN, bulbar, or respiratory involvement
	Syringomyelia	Dissociated sensory loss, slow progression, younger population
	B12 deficiency	Prominent sensory findings
Anterior horn	Post-poliomyelitis syndrome	History of paralytic poliomyelitis, paucity of UMN signs and slow rate of progression
	Spinal muscular atrophy	Slowly progressive, symmetrical, proximal muscle weakness and atrophy without additional UMN signs
	Monomelicamyotrophy	Young men in their second and third decades, relatively sparse fibrillation on needle EMG
Neuropathies and plexopathies	Multifocal motor neuropathy	Absence of muscle atrophy despite very significant weakness, motor weakness is typically restricted to multiple separate peripheral motor nerves, anti GM1
	Neuralgic amyotrophy	preceded by significant deep, aching pain, involvement of motor nerve fibers can be curiously patchy
Disorders of the neuromuscular junction	MG	Absence of UMN signs and fasciculations, absence of fibrillation and fasciculation on needle EMG
Myopathies	IBM	Absent fasciculations, no UMN signs
	Oculopharyngeal muscular dystrophy	Involvement of eyelids and extraocular muscles
	Isolated neck extensor myopathy	The weakness does not spread to other regions

EMG: Electromyography; LMN: Lower motor neuron; UMN: Upper motor neuron; IBM: Inclusion body myositis; MG: Myasthenia gravis; VLCFA: Very long chain fatty acids; SNAPs: Sensory nerve action potential; Hex: Hexosaminidase; EDX: Electrodiagnostic; PNS: Peripheral nervous system; CNS: Central nervous system

## Conclusion

Although the essential diagnostic criteria of ALS are defined by the El Escorial criteria, there are still many misdiagnosis. Our misdiagnosis of ALS mainly relates to diagnostic difficulty, and, also to lack of skill and knowledge about MNDs. To reduce the misdiagnosis rate, enhanced knowledge of the potential alternative disease and MND diagnostic pitfalls are essential, particularly, if the key points are considered.

The differential diagnosis should rule out non-motor neuron similar diseases, especially treatable conditions and other adult-onset MND with limited or focal presentations (Table 4).

**Table 4:** Diagnosing amyotrophic lateral sclerosis (ALS)/ motor neuron diseases (MND): recommended investigations^37^

**Clinical chemistry**			
**Test**	**Evidence ** **class**	**Recommended ** **mandatory tests**	**Recommended additional ** **tests in selected cases**
Blood			
Erythrocyte sedimentation rate	IV	×	–
C-reactive protein	IV	×	–
Hematological screen	IV	×	–
AST, ALT, LDH	IV	×	–
Thyroid function test	IV	×	–
Vitamin B_12_ and folate	IV	×	–
Serum protein electrophoresis	IV	×	–
Serum immunoelectrophoresis	IV	×	–
CK	IV	×	–
Creatinine	IV	×	–
Electrolytes (Na^+^, K^+^, Cl^−^, Ca^2+^)	IV	×	–
Glucose	IV	×	–
Angiotensin-converting enzyme	IV	–	×
Lactate	IV	–	×
Hex A and B assay	IV	–	×
Ganglioside GM-1 antibodies	IV	–	×
Anti-Hu, anti-MAG	IV	–	×
RA, antinuclear antibodies, anti-DNA	IV	–	×
Anti-acetylcholine receptor and anti-muscle-specific receptor tyrosine kinase antibodies	IV	–	×
Serology (Borrelia, virus including HIV)	IV	–	×
DNA analysis (for SOD1, SMN, SBMA, TDP43, FUS)	IV	–	×
CSF			
Cell count	IV	–	×
Cytology	IV	–	×
Total protein concentration	IV	–	×
Glucose, lactate	IV	–	×
Protein electrophoresis including IgG index	IV	–	×
Serology (Borrelia, virus)	IV	–	×
Ganglioside antibodies	IV	–	×
Urine			
Cadmium	IV	–	×
Lead (24-h secretion)	IV	–	×
Mercury	IV	–	×
Manganese	IV	–	×
Urine immunoelectrophoresis	IV	–	×
Neurophysiology			
Electromyography	III	×	–
Nerve conduction velocity	III	×	–
tcMEP (TMS)	IV	–	×
Radiology		–	–
MRI/computed tomography (cranial/cervical, thoracic, lumbar)	IV	×	–
Chest X-ray	IV	×	–
Mammography	IV	–	×
Biopsy			
Muscle	III	–	×
Nerve	IV	–	×
Bone marrow	IV	–	×
Lymph node	IV	–	×

ASAT: Aspartate aminotransferase, ALAT: Alanine aminotransferase; LDH: Lactate dehydrogenase; CK: Creatine kinase; MAG: Myelin-associated glycoprotein; RA: Rheumatoid arthritis; Hex: Hexosaminidase; HIV: Human immunodeficiency virus; SBMA: Spinobulbar muscular atrophy; SMN: Survival of motor neuron; SOD1: Superoxide dismutase 1; FUS: Fused in sarcoma; TMS: Transcranial magnetic stimulation; MEP: Motor-evoked potentials; IgG: Immunoglobulin G, MRI: Magnetic resonance imaging

## Notes:

**How to cite this article:** Ghasemi M. Amyotrophic lateral sclerosis mimic syndromes. Iran J Neurol 2016; 15(2): 85-91.
